# An Update on Protein Kinases as Therapeutic Targets—Part I: Protein Kinase C Activation and Its Role in Cancer and Cardiovascular Diseases

**DOI:** 10.3390/ijms242417600

**Published:** 2023-12-18

**Authors:** Shmuel Silnitsky, Samuel J. S. Rubin, Mulate Zerihun, Nir Qvit

**Affiliations:** 1 The Azrieli Faculty of Medicine in the Galilee, Bar-Ilan University, Henrietta Szold St. 8, Safed 1311502, Israel; shmuel.silnitsky@biu.ac.il (S.S.); mulate.zerihun@biu.ac.il (M.Z.); 2 Department of Medicine, School of Medicine, Stanford University, 300 Pasteur Drive, Stanford, CA 94305, USA; yrubin@stanford.edu

**Keywords:** protein kinase, protein kinase C (PKC), cancer, cardiovascular disease, allosteric, peptides

## Abstract

Protein kinases are one of the most significant drug targets in the human proteome, historically harnessed for the treatment of cancer, cardiovascular disease, and a growing number of other conditions, including autoimmune and inflammatory processes. Since the approval of the first kinase inhibitors in the late 1990s and early 2000s, the field has grown exponentially, comprising 98 approved therapeutics to date, 37 of which were approved between 2016 and 2021. While many of these small-molecule protein kinase inhibitors that interact orthosterically with the protein kinase ATP binding pocket have been massively successful for oncological indications, their poor selectively for protein kinase isozymes have limited them due to toxicities in their application to other disease spaces. Thus, recent attention has turned to the use of alternative allosteric binding mechanisms and improved drug platforms such as modified peptides to design protein kinase modulators with enhanced selectivity and other pharmacological properties. Herein we review the role of different protein kinase C (PKC) isoforms in cancer and cardiovascular disease, with particular attention to PKC-family inhibitors. We discuss translational examples and carefully consider the advantages and limitations of each compound (Part I). We also discuss the recent advances in the field of protein kinase modulators, leverage molecular docking to model inhibitor–kinase interactions, and propose mechanisms of action that will aid in the design of next-generation protein kinase modulators (Part II).

## 1. Introduction

Protein kinases are a large and diverse family of more than 500 proteins encoded by ~2% of the human genome, classified into eight superfamilies (AGC, aPK, CAMK, CMGC, STE, TK, TKL, and CK1) according to homology within their catalytic domains. Kinases catalyze phosphorylation, the chemical addition of a phosphoryl group (PO_3_^−^) to the target substrate by the hydrolysis of a γ-phosphate group from adenosine triphosphate (ATP), a reversible and the most widespread type of post-translational modification (PTM) used in signal transduction. In eukaryotic organisms, protein phosphorylation primarily occurs on serine, threonine, and tyrosine residues. Although sugars and lipids can also be phosphorylated, here we are focusing on protein phosphorylation. Approximately one-third, and up to two-thirds, of the proteins in a cell may be phosphorylated at one time or another, affecting a very large set of cellular pathways by turning activities “on” or “off” [1]. Phosphorylation plays major roles in numerous cellular functions, including transcription, translation, metabolism, proliferation, division, cell-cycle progression, biosynthesis, movement, and survival. These processes are critical to cellular homeostasis, and dysregulated kinase activity has been linked to a variety of pathological conditions, such as neurodegeneration [2], inflammation [3], autoimmunity [3], cancer [4,5], and cardiovascular diseases (CVDs) [6]. Furthermore, protein phosphorylation is significantly underlined by the Nobel Prize in Physiology or Medicine (1992) awarded to Edmond H. Fischer and Edwin G. Krebs “for their discoveries concerning reversible protein phosphorylation as a biological regulatory mechanism”. Their major contribution was the discovery that the conversion of the inactive enzyme phosphorylase b to active phosphorylase a is caused by phosphorylation, which is regulated by the protein kinase called phosphorylase kinase [7,8]. Given this importance, it is not surprising that protein kinases are the second most therapeutically targeted group of proteins, after the G-protein-coupled receptors (GPCRs) [9,10,11,12], and the pharmaceutical industry has dedicated approximately one-third of new drug development programs over the last decade to the development of protein kinase modulators [13,14].

Imatinib (i.e., STI571, or Gleevec), which received Food and Drug Administration (FDA) approval in 2001, is considered the first protein kinase inhibitor that was clinically approved [15]. Although fasudil (an inhibitor of Rho-dependent kinases) was approved in 1995, and rapamycin (i.e., sirolimus, an inhibitor of the protein kinase TORC1) was approved in 1999, both were approved without knowledge of the identity of their target proteins. Imatinib is an oral treatment for chronic myelogenous leukemia (CML), an uncommon type of cancer of the bone marrow, which arises from a pluripotent hematopoietic stem cell that has undergone a reciprocal translocation between the breakpoint cluster region protein (Bcr) gene on chromosome 22 and the Abelson tyrosine-protein kinase 1 (Abl1) proto-oncogene on chromosome 9. The fusion Bcr–Abl protein results in a shortened chromosome 22 (i.e., the Philadelphia chromosome) that lacks the autoinhibitory domains of the Abl1 kinase and, therefore, results in the constitutive activation of Abl1 proliferative function. Imatinib was developed in the late 1990s by the biochemist Nicholas Lyndon, and its use to treat CML was driven by Brian Druker. The drug is a potent small-molecule kinase inhibitor (SMKIs) that functions as a competitive inhibitor of the ATP binding site that interacts with the Abl1 domain via six hydrogen bond interactions, leading to a stabilized imatinib–Bcr–Abl complex; this prevents ATP from reaching its binding site, resulting in inhibition of the Bcr–Abl tyrosine kinase [16,17]. Imatinib has revolutionized drug therapy for CML, and the drug was featured on the front cover of Time Magazine (28 May 2001, Vol. 157 No. 21) and termed a “magic bullet”. Lyndon, Druker, and their colleagues were granted the Lasker–DeBakey Clinical Medical Research Award in 2009 for “converting fatal cancer into a manageable condition”. Imatinib also became one of the world’s most commercially successful drugs with peak sales of USD 4.6 billion in 2012. The spectacular efficacy of Imatinib was observed as soon as it entered clinical trials in 1998. A large-scale phase III trial, the international randomized study of interferon and STI571 (IRIS), compared a single daily dose of imatinib to interferon alpha (IFNα)/cytarabine treatment in newly diagnosed CML patients and showed the superior efficacy of imatinib over IFNα/cytarabine (76% versus 15%, respectively) [18], a five-year survival rate of 85% [19], and enormous commercial success. This changed the perception of protein kinases as drug targets, which had previously received skepticism from many pharmaceutical companies.

Since imatinib, a plethora of targeted therapeutic agents have been developed and tested in humans, and many of these trials, predominantly those targeting mitogen-activated protein kinase (MAPK) signaling, have demonstrated significant improvement versus the standard of care and resulted in FDA approvals [20,21]. Currently, there are 98 approved kinase inhibitors worldwide, 71 of which are SMKIs that have been approved by the FDA, targeting 21 kinase families constituting approximately 20% of the kinome. Interestingly, the number of SMKIs approved by the FDA has more than doubled since 2016, with 37 new approvals, making SMKIs approximately 15% of all novel drug approvals in the last 5 years (2016–2021). In addition, 16 more SMKIs have been granted approval by other regulatory agencies [22].

Most approved SMKIs are indicated for oncologic conditions. The lack of SMKI selectivity is the main reason why there has been less success in producing kinase-inhibitor drugs for other conditions [23]. Nevertheless, SMKIs have been approved for several other diseases. For example, Janus kinase (JAK) inhibitors (e.g., ruxolitinib, tofacitinib, baricitinib, upadacitinib, and fedratinib) have been approved for the treatment of autoimmune diseases, inflammatory disorders, and dermatoses [24]. Rho-associated protein kinase (ROCKs) inhibitors (e.g., netarsudil and ripasudil) have also been approved for various conditions related to primary open-angle glaucoma (POAG) and other etiologies of increased intraocular pressure [25,26]. The number of SMKIs that are being approved for the treatment of diseases in nononcologic therapeutic fields continues to rise, and, currently, one-third of the SMKIs in clinical development aim to address disorders beyond oncology [13,24,27,28].

The pharmacological modulation of kinase activity is almost exclusively achieved through SMKIs that target the conserved kinase domain. Attaining the selectivity required for SMKIs to become successful drugs remains one of the largest hurdles in an early-stage kinase-inhibitor project. Certain disease processes (e.g., oncology) that are a result of multiple aberrant pathway activation may still benefit from the use of multikinase inhibitors that target several pathways at the same time (e.g., lenvatinib and sorafenib multikinase inhibitors approved for the treatment of progressive radioiodine-refractory differentiated thyroid cancer). Yet, such compounds still need to be selective inhibitors of a well-defined set of kinase enzymes, and the disadvantage of this approach may be the increase in adverse effects that comes with inhibiting multiple signal-transduction pathways, which can also be critical to physiological functions. Therefore, the lack of selectivity of multikinase inhibitors causes, in many cases, toxicities that result in the discontinuation of promising drug candidates [29,30,31]. Currently, the majority of the FDA-approved kinase inhibitors are SMKIs targeting the kinase ATP-binding site (63 SMKIs) [32]. However, in many cases, these SMKIs demonstrate low specificity towards the target kinase, resulting in problems with toxicity and a variety of side effects. Therefore, the pharmaceutical industry continues to invest in emerging trends focused on identifying alternative approaches to specifically target protein kinases more selectively.

One rapidly emerging technology leverages proteolysis-targeting chimera (PROTAC) molecules, which harness the ubiquitin–proteasome system to degrade a target protein and was first proposed by the pioneers Crews and coworkers in 2001 [33] (for recent reviews [34,35,36]). Since its first report, the technology has expanded from academia to industry. To date, PROTACs are being validated by several programs in preclinical and early clinical development, and the first oral PROTACs (ARV-110 and ARV-471) have shown encouraging results in clinical trials for prostate and breast cancer treatment [37]. Another exciting approach involves the use of antibodies, which can provide superior potency, selectivity, and pharmacokinetic properties compared to small molecules [38]. Indeed, several antibodies have been approved, such as cetuximab in colorectal and head and neck cancer, and trastuzumab in breast cancer [39,40,41]. However, both PROTACs and antibodies also demonstrated some inherent challenges. PROTACs are more difficult and more expensive to develop than SMKIs, they do not readily degrade many types of proteins, their toxicology is uncertain, their adverse effects are largely unknown, and their oral bioavailability is low [37,42,43]. Antibodies demonstrate several challenges as well, including production cost, tissue accessibility, and immunogenicity [41,44,45,46]. Since the majority of SMKIs target the ATP site that is structurally similar, many of them suffer from a lack of selectivity for structurally related kinase families, resulting in off-target toxicity that can cause dangerous side effects and is a major cause of clinical trial failure [14,47,48].

Protein kinase C (PKC) is a prototypical class of serine/threonine kinases within the AGC-kinase superfamily named after protein kinase A, cyclic GMP-regulated kinases, and PKC. The AGC-kinase superfamily consists of 63 evolutionarily related serine/threonine protein kinases that are divided into 14 families and 21 subfamilies. PKC was originally identified by Nishizuka and colleagues over 40 years ago as a phospholipid and calcium-dependent protein kinase that phosphorylated histone and protamine. Originally, the enzyme was called protein kinase M (PKM) since only magnesium was required for activity. In subsequent studies, it was discovered that PKM was the proteolytic product of an enzyme whose activity was stimulated by Ca^2+^. This enzyme was named PKC because it was activated by second-messenger Ca^2+^ [49,50]. PKCs play a vital role in signal transduction by phosphorylation of target proteins to control numerous cellular functions. The PKC family of mammals consists of nine different genes and includes a variety of members due to alternative splicing [51,52]. Based on their structural components and activation mechanisms, PKCs are subdivided into three groups: classical PKC, novel PKC, and atypical PKC. The complexity in PKC signaling arises from the fact that PKC is a superfamily of structurally related kinases with diverse biological functions and, in some cases, opposing roles in cell proliferation, differentiation, apoptosis, and angiogenesis [53,54]. Precise control of PKC signal amplitude is necessary for the healthy condition of a cell and an organism, and the alteration of normal PKC activity is linked to numerous human diseases, including metabolic disorders [55,56], neurodegenerative disease [2], autoimmune disease [57,58,59,60], cancer [61], and CVDs [62,63,64]. Therefore, the development of selective PKC modulators is predicted to represent a breakthrough in drug discovery and has become the subject of intense studies by academic laboratories and pharmaceutical companies [65,66,67,68]. Unraveling the enormous complexity of the mechanisms by which PKC isozymes impact human diseases is key for assessing their potential as therapeutic targets.

A selection of reviews on the protein kinase family and on protein kinase modulators have been published recently. Several reviews from a basic research perspective featured critical advances in understanding kinase signal-transduction pathways [69,70,71,72]. Other reviews focused on the advantages of targeting protein kinases in treating various human diseases [14,73,74,75,76], such as immune disorders [77,78], cancer [71,79], CVDs [6], and other associated conditions [24,80]. Finally, several reviews focused on the FDA and other regulatory approvals of kinase inhibitors [76,81] and targets in clinical trials [13]. To the best of our knowledge, there is not an up-to-date review published on kinase allosteric modulators nor the role of peptides in therapeutic applications targeting PKC. In these two reviews, we first provide an updated summary of the current literature about PKC’s role in human diseases, emphasizing its critical role in cancer and CVDs (Part I). Next, we aim to provide insight into alternative therapeutic approaches developed to target PKC and highlight the opportunities and challenges in applying these advances to cancer and CVDs. Furthermore, using available structures, the AlphaFold protein-structure database [82,83], and computational methods, we leverage molecular docking to model the interaction of previously developed modulators and explore their mechanisms of action. We discuss examples of successful applications and insights into the strengths and limitations of each modulator, thus providing novel tools and guidance to new and established researchers in the field (Part II).

## 2. The Protein Kinase C (PKC) Family

PKC is a ubiquitous enzyme found in almost all human cell types including the endothelium, vascular smooth muscle, and fibroblasts. The PKC family comprises a group of highly related protein kinases that phosphorylate serine and threonine residues on a large number of proteins and therefore regulate diverse cellular responses. PKC mediates significant and sometimes opposing effects in different tissues and is widely implicated across physiological and pathological processes [84]. The members that populate the mammalian PKC family evolved from the single PKC in saccharomyces cerevisiae, PKC1 [85,86]. PKC isozymes have been classified into three groups, ‘conventional’ or ‘classical’ PKCs (cPKCs) that are composed of PKCα, two splice variants of PKCβ (PKCβI and PKCβII), and PKCγ, which all require phosphatidylserine (PS), diacylglycerol (DAG), and Ca^2+^ to be activated; ‘novel’ PKCs (nPKCs), a group that includes PKCδ, PKCε, PKCη, and PKCθ, which require only PS and DAG to be activated; and ‘atypical’ PKCs (aPKCs), including PKCζ and PKCι/λ, which require only PS.

**Figure 1:**
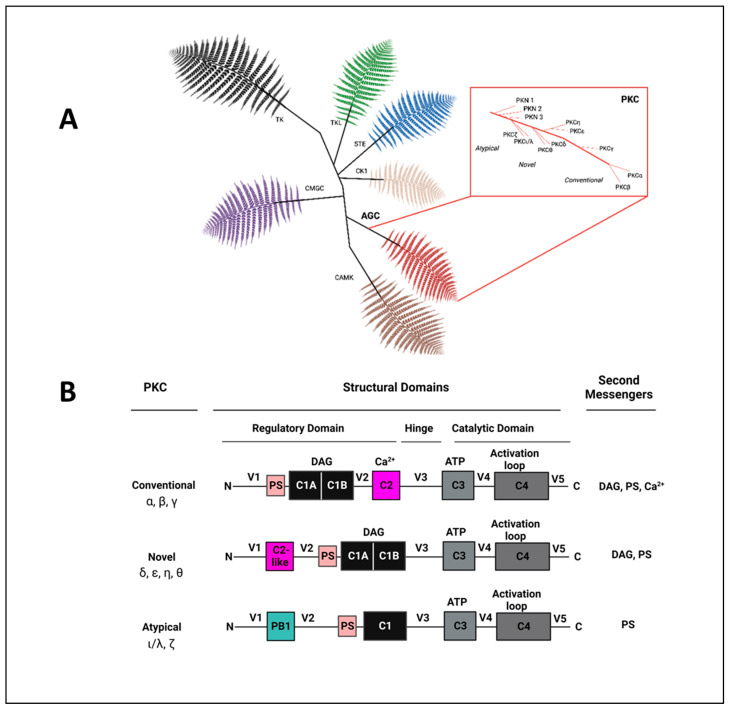
PKC isozyme diversity. (**A**) The human kinome (adapted from www.cellsignal.com/reference/kinase) phylogeny is extensive, and the PKC family is one member of the AGC superfamily (bottom right), with three groups of conventional, novel, and atypical PKC isozymes. (**B**) PKC isozymes are homologous but contain a distinct set of structural domains responsible for their diverse functions and interactions with second messengers and other binding partners. All PKC family members are constituted by four conserved domains (C1–C4) separated by a hinge region. The pseudo-substrate site (PS) keeps the protein in its inactive form. Second messengers are indicated on the right side of the picture. BioRender.com was used to generate this figure.

All family members share a common architecture of an amino-terminal regulatory moiety (approximately 35 kDa) linked by a flexible hinge segment to a carboxyl-terminal kinase domain (approximately 45 kDa). PKC comprises a single polypeptide structure consisting of a regulatory domain and a catalytic (kinase) domain separated by a hinge region (V3) with four functional conserved regions (C1–C4), which are interspersed by five variable regions (V1–V5). The regulatory domain comprises C1 and C2 regions that interact with DAG, PS, and Ca^2+^, thereby acting as the membrane-targeting module. The catalytic domain comprises the C3 and C4 domains that constitute the ATP- and substrate-binding lobes of the kinase core. Conventional isozymes contain tandem C1 domains (C1A and C1B) that bind to DAG or phorbol esters found in cell membranes, as well as a C2 domain that binds membranes in the presence of the second-messenger Ca^2+^. Novel PKC isoenzymes similarly possess two tandem C1 domains that bind to DAG or phorbol esters. Interestingly, the C1B domain of novel PKCs has a 100-fold higher affinity for DAG compared with the C1B domain of classical PKCs. Novel PKCs also contain a variant of the C2 domain (novel C2 domain), which lacks key residues that interact with Ca^2+^. To compensate for the lack of C2 domain contribution to membrane recruitment, novel PKC activation only requires DAG [87,88]. Atypical PKC isozymes contain a single atypical C1 domain that retains the ability to bind anionic phospholipids, as well as a Phox and Bem1 (PB1) domain that mediates protein–protein interactions (PPIs). While the PKC isozyme catalytic domains are very similar, they differ from each other in the C2 region of the regulatory domain as well as in the variable regions. The regulatory domain of all PKC isoenzymes contains a short autoinhibitory pseudo-substrate sequence whose occupation of the kinase substrate-binding cavity maintains these kinases in an inactive state [54,89]. While both catalytic and regulatory domains represent target opportunities for the development of novel therapeutics to modulate PKC activity, each domain also presents its own challenges for achieving selective compounds (Figure 1).

PKC activation relies on three molecular mechanisms: (i) phosphorylation, (ii) cofactor binding, and (iii) intracellular translocation. Like many other kinases, PKC is regulated by phosphorylation, which can alter enzyme activity, regulate protein stability, affect protein interactions or localization, and influence other PTMs. For PKC to become activated, the protein must first undergo phosphorylation on several critical residues, which occurs shortly after biosynthesis via a process called PKC priming. Newly synthesized isozymes are in an open and degradation-sensitive confirmation until they undergo a sequence of stabilizing phosphorylations on several conserved residues, resulting in a stable, autoinhibited enzyme that is primed to respond to second messengers. Conventional and novel PKCs are constitutively phosphorylated at three conserved domains: the activation loop, the turn motif, and the hydrophobic motif. Atypical PKCs are phosphorylated at the activation loop and turn motif but contain a phosphomimetic glutamic acid at the hydrophobic motif. Additionally, several PKC isozymes require ERK activation to exert important downstream effects.

Mass spectrometric studies of the conventional PKCβII isozyme identified three constitutively phosphorylated sites located in the PKC kinase domain (Thr500 at the activation loop, Ser641 at the turn motif, and Ser660 at the hydrophobic motif); interestingly, these phosphorylation sites are highly conserved among most AGC kinases, including protein kinase B (PKB, or Akt), in addition to PKC [90,91]. In order for PKC to undergo priming phosphorylation, conventional and novel PKCs bind to the chaperone heat-shock protein 90 (Hsp90) and its cochaperone cell-division cycle 37 (Cdc37), which promotes the maturation of these kinases by acting as an adapter and loading them onto the Hsp90 complex; the complex then binds open PKC via a conserved PXXP motif in the C-terminal tail that harbors a conserved phosphorylation site termed the hydrophobic motif [92]. Next, phosphoinositide-dependent kinase-1 (PDK-1, a key regulator of PKCs) phosphorylates amino acids in the activation loop (e.g., Thr500 in PKCβII). Phosphorylation by PDK-1 is likely the first phosphorylation event in the processing of PKC by phosphorylation. This phosphorylation makes a conformational change that triggers two rapid subsequent phosphorylation events at the carboxy terminus. First occurs phosphorylation of the turn motif that, in many cases, is regulated by the mammalian target of rapamycin complex 2 (mTORC2) [93,94], followed by phosphorylation at the hydrophobic motif (e.g., Thr641 and Ser660 in PKCβII) [95]. The phosphorylation of the turn motif stabilizes the structure of mature PKC by anchoring the carboxyl-terminal tail on the upper lobe of the kinase to stabilize the active conformation of the kinase. Finally, PKC autophosphorylates by an intramolecular reaction at the hydrophobic motif, which is regulated by the interaction of Hsp90 with the PXXP clamp. The result is a stable PKC that is released into the cytosol in a catalytically competent closed conformation (i.e., autoinhibited state) in which the pseudo-substrate region masks the substrate-binding cavity.

**Figure 2:**
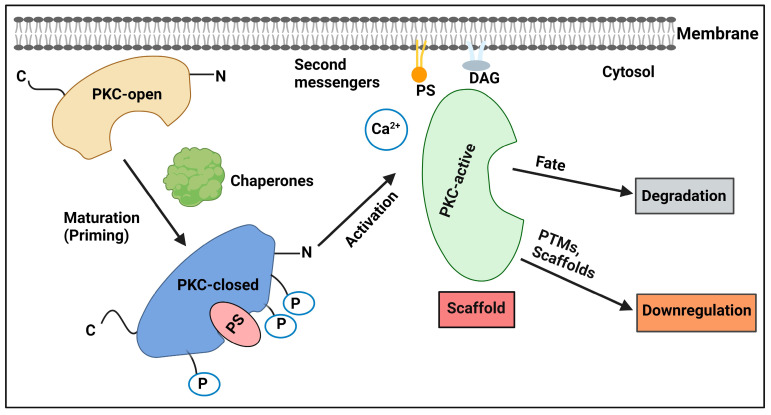
PKC maturation, activation, and cytosolic fate. The open PKC matures via a priming process involving three critical phosphorylation events, which is facilitated by chaperone complex proteins. PKC adopts a closed, autoinhibited, conformation in which the pseudo-substrate (PS) domain is trapped in the substrate-binding site, and the primed enzyme localizes to the cytosol. The closed mature PKC in the cytosol is then activated by second messengers and scaffold proteins that mediate the target specificity of the kinase. The cytosolic survival of the species and the fate of the protein are determined by other scaffolds and post-translational modifications that govern downregulation and degradation reactions. BioRender.com was used to generate this figure.

Each phosphorylation event induces conformational changes in the PKC molecule that result in altered thermal stability, resistance to phosphatases, and catalytic competency. Phosphorylation is vital to render PKC in a catalytically competent conformation and to protect PKC from degradation. Mechanisms that prevent the phosphorylation of PKC, such as the loss of PDK-1, inhibition of mTORC2, or impairment of PKC’s intrinsic catalytic activity, prevent the maturation of PKC, resulting in PKCs that are highly susceptible to degradation. Therefore, these steps play a critical role in controlling cellular levels of PKC [93,94,96,97]. Since PKC phosphorylation is constitutive, its activity is regulated by the cellular levels of PKC, which are directly regulated by its phosphorylation. Understanding how to modulate these levels has important therapeutic implications, as altered PKC levels correlate with various human diseases [98,99,100,101]. In addition to the described phosphorylation sequence, PKC has an abundance of other PTMs, such as additional phosphorylation events, acetylation, and ubiquitination [100,102,103]. Whether any of these or other yet-to-be-identified sites are critical for PKC “priming” and maturing remains to be established (Figure 2).

### 2.1. Regulation by Lipid Second Messengers

The fully phosphorylated “mature” PKC is localized to the cytosol in an autoinhibited and stable conformation that is poised to respond to second messengers. Lipid second messengers are signaling molecules produced in response to extracellular stimuli and coordinate signaling by recruiting and activating kinases. Although after phosphorylation PKC is catalytically competent, an autoinhibitory pseudo-substrate that binds the substrate-binding cavity maintains PKC inactive until the appropriate second messengers bind. The activity of mature conventional and novel PKC isozymes is regulated by binding to second messengers, such as DAG and Ca^2+^. Yet, the differential binding of second messengers between conventional PKCs (DAG and Ca^2+^) and novel PKCs (solely DAG, as they are not responsive to Ca^2+^) leads to substantial differences in their spatiotemporal signaling dynamics.

Two prominent lipid second-messenger pathways are those mediated by DAG and phosphatidylinositol (PI), a minor phospholipid with a characteristic fatty acid profile. Initially, extracellular signals cause the hydrolysis of phosphatidylinositol-4,5-bisphosphate (PIP2), DAG is generated, and Ca^2+^ is released from intracellular stores. Next, the binding of Ca^2+^ to the C2 domain recruits conventional PKC isozymes primarily to the plasma membrane. Ca^2+^ binding induces a conformational change in which the C2 domain is displaced from the kinase domain, exposing a PIP2-binding basic face that is masked in the autoinhibited conformation, resulting in the localization of PKC to the plasma membrane. The C1B domain of inactive PKC also binds to its membrane-embedded ligand, DAG. The coordinated engagement of both the C1 and C2 domains on the membrane provides the energy, prompting a second conformational change that expels the autoinhibitory pseudo-substrate from the substrate-binding cavity and reveals an active open confirmation, allowing substrate phosphorylation. Novel PKC isozymes are activated by DAG alone, allowing them to be activated by phospholipase C-catalyzed hydrolysis of lipids other than PIP2. Since novel isozymes do not have a functional C2 domain but have a C1A domain that has a 100-fold higher affinity for DAG, they are translocated mainly to DAG-rich endomembranes such as the Golgi. Atypical PKCs are regulated by neither DAG nor calcium, but they are regulated by the PPIs of their PB1 domain to the PB1 domains of protein scaffolds, such as p62 and Par6. After binding to these proteins, atypical PKC isozymes are harbored near the plasma membrane in proximity to their substrates. Furthermore, in many cases, this interaction moves the pseudo-substrate domain away from the substrate-binding cavity, thus relieving autoinhibitory constraints [104].

Conformational changes in all PKC isoforms allow the proteins to interact with specific anchoring and/or scaffold proteins, which determine intracellular translocation and functional activity. Tsien et al. pioneered technologies for the development of fluorescence resonance energy transfer (FRET) reporters to measure the spatiotemporal dynamics of PKC signaling in live cells [105], which revealed that Ca^2+^ oscillations, with or without DAG oscillations, drive oscillations of conventional PKC activity [106,107]. Live-cell imaging studies demonstrated that there are different “signatures” of PKC activity at defined sublocations in cells [108]. For example, rapid rises in intracellular Ca^2+^ drive rapid activation of conventional PKC isozymes at the plasma membrane [109], while relatively high levels of DAG (e.g., in the Golgi membranes) mediates a “basal” interaction of novel PKC isozymes at the Golgi [110].

Interestingly, the PKC enzyme superfamily exhibits an especially high degree of complexity and variability, as specific conventional and novel PKCs can also be activated independently of second messengers. Certain PKCs can be activated by the accumulation of reactive oxygen species (ROS), which are elevated in various human disease processes, including neurodegeneration, cancer, and CVDs [111,112]. For example, PKCδ is modified at multiple tyrosine residues by Src-dependent phosphorylation in the regulatory moiety in response to cell stimulation with H_2_O_2_, inducing DAG-independent activation and effects on localization [113,114,115]. H_2_O_2_-induced Tyr311 phosphorylation has also been proposed to activate PKCδ by inducing caspase-3 cleavage between its regulatory and catalytic domains, resulting in a nuclear-localized uninhibited catalytic domain [116]. In addition, the PKCδ nuclear localization sequence (NLS) binds importin-α, which facilitates PKCδ transport through the nuclear pore complex [115]. Thus, in addition to reversible activation by binding second messengers, alternative mechanisms of activation fine-tune the localization, function, and activity of individual PKC isozymes, depending on their microenvironments.

### 2.2. Regulation by Scaffold Interactions

A critical mechanism to control protein kinase specificity relies on scaffold and/or anchored proteins. As multiple PKC isozymes are expressed in the same cell and respond to the same stimuli (e.g., the same second messenger), specificity in their localization and activity is mediated also by protein scaffolds. Scaffolds are a group of proteins that coordinate and allow specificity and fidelity by compartmentalizing kinases and their downstream substrates. These proteins provide ways to mediate the action of promiscuous enzymes by controlling their access to particular substrates (e.g., an anion channel in the cell membrane vs. a promoter of gene expression in the nucleus [117,118]).

The best-characterized class of kinase scaffolds is the A-kinase anchoring proteins (AKAPs) that compartmentalize protein kinase A (PKA). AKAPs were first identified as PKA protein scaffolds [119] and were demonstrated to play a critical role in signal transduction, placing binding effectors into specific subcellular locations, and optimizing signaling events on colocalized enzymes, receptors, and mRNA molecules to maintain cell homeostasis [120,121]. Protein scaffolds also have an integral role in PKC signaling, as they play a critical role in determining PKC isozyme localization. Furthermore, these protein scaffolds are vital in establishing substrate specificity [122,123,124]. Initial PPI studies by Mochly-Rosen and colleagues identified several PKC anchoring proteins, collectively termed receptors for activated C kinases (RACKs), which were shown to target a different PKC isozyme [125] (for reviews, see [126,127,128]). Over the years, additional protein scaffolds were identified, including 14-3-3 [129], annexins [130], HSPs [131], importins [132], and AKAPs that universally bind PKA [121]. While AKAPs have since been shown to interact with both PKA and PKC, PKC binding occurs through regions that are distinct from that of PKA anchoring [133,134].

RACKs bind to activated PKC isozymes in a selective and saturable manner, anchoring the PKC isozymes to their respective subcellular sites. For example, RACK1 (i.e., guanine nucleotide-binding protein subunit beta-2-like 1, or GNB2L1) is selective for PKCβII [135], and RACK2 (previously identified as β‘COP’ [136,137]) is selective for PKCε [138]. In general, RACKs are present in a particular location, their binding to PKC is dependent on a second messenger, PKC binding to RACKs is saturable and specific, and RACKs are neither a PKC substrate nor an inhibitor [139]. Both RACK1, which is selective to PKCβII, and RACK2, which is selective to PKCε, are members of the tryptophan-aspartate (WD) repeat family, containing seven repeats of the WD40 motif. The Mochly-Rosen laboratory not only identified and characterized several RACKs but also engineered peptides derived from scaffold proteins that resemble domains in specific PKC isozymes and proposed that these sequences participate in intramolecular interactions that clamp PKC in an autoinhibited state [140]. They hypothesized that peptides derived from these sequences disrupt the PPIs of specific PKC isozymes with their cognate RACKs and, therefore, modulate the cellular function of PKC isozymes [141]. Such peptides have been particularly valuable pharmacological tools to regulate the activity of PKC isozymes in cancer [142] and CVDs [143,144]. We further discuss these peptides, feathering their rational design and bioactivity vide infra. Another interesting example of the role of protein scaffolds in coordinating PKC activity was contributed by Zuker and colleagues, who demonstrated that the disruption of the interaction between the eye-specific PKC in *Drosophila melanogaster* and the PDZ scaffold InaD impairs light signaling [145]. Currently, it is well established that scaffold PPIs are an integral and vital part of PKC regulation, poising specific isozymes near key protein and lipid regulators as well as substrates. Therefore, disruption of these critical PPIs presents a unique avenue for specifically tuning PKC output without the inhibition of catalytic activity to reduce adverse effects (Figure 2).

## 3. PKC in Cancer

Individual cells constitute the fundamental units of life with a specific physiological purpose in different organs and organ systems. Under physiological conditions, cells would divide in a coordinated and controlled process, maintaining a homeostatic environment in the body. However, in pathological conditions, some cell types can become unresponsive to the cell-division regulatory signals and start to proliferate aberrantly; this clinical situation is most frequently termed cancer, although there are specific terms to describe the exact type of cellular growth dysregulation (hyperplasia, metaplasia, dysplasia, neoplasia, etc.). Cancer is a dynamic process that evolves in response to both genetic and epigenetic alterations, a highly challenging disease due to heterogeneity and robust compensatory mechanisms that modify cellular signaling pathways, giving rise to multiple cellular populations that confer resistance either de novo or in response to treatment. Hundreds of protein kinases play overlapping roles in cancer behavior, including cell transformation, tumor initiation, tumor progression, survival, proliferation, and recurrence. Kinases are associated with cancer via dysregulated gene expression and/or amplification, gene mutation, chromosomal translocation, epigenetic modification, and aberrant protein phosphorylation. Protein kinase dysfunction is implicated in multiple different components of carcinogenesis. Furthermore, protein kinases are among the early favored targets for cancer therapy. The development of protein kinase inhibitors (PKIs) has been a significant breakthrough in targeted cancer therapy, driving strategies against specific cancer-signaling pathways. Since the early success of the first SMKI, imatinib, which led to a paradigm shift in cancer therapy, the treatment of numerous additional cancers has proven clinically successful with kinase modulators. Many comprehensive reviews provide an overview of kinase-targeted drug discovery and development in relation to oncology, discussing the challenges, mechanisms of inhibition, outcomes, adverse effects, and future potential for kinase-targeted cancer therapies [13,14,31,74,146,147]. Currently, there are 58 agents targeting different protein kinases approved by the FDA for the treatment of cancer; 49 of these modulators are indicated for solid tumors including breast, lung, and colon tumors; 5 are indicated for nonsolid tumors, such as leukemias; and 4 (e.g., acalabrutinib, ibrutinib, imatinib, and midostaurin) are indicated for both solid and nonsolid tumors. Despite these encouraging results, problems with drug resistance and toxicity present major challenges for both clinical and experimental oncology [76,148].

The recognition of PKC as the long sought-after receptor for the tumor-promoting phorbol esters established the potential role of PKC in carcinogenesis and as a highly valuable target in oncology [149,150,151]. Phorbol esters (i.e., tetracyclic diterpenoids) are natural compounds derived from the plants of the family *Euphorbiaceae*, which mimic the action of the lipid second-messenger DAG by binding to the PKC C1 domain in a competitive manner. Early studies starting in the 1940s established that phorbol esters have potent activity as skin tumor promoters in mice [152]. However, unlike DAG, they are not readily metabolized and thus lead to constitutive activation of PKC, resulting in chronic loss, or downregulation, of PKC. The general assumption is that, upon phorbol ester activation, all classic and novel PKCs will be activated and phosphorylate specific substrates [153]. Moreover, there is an abundance of cancer-associated mutations in PKC that are loss-of-function (LOF) mutations and are found in a multitude of cancers. There are over 1000 cancer-associated somatic mutations in PKC isozymes identified to date. There are about 20–25% PKC mutations in several cancers, including melanoma, colorectal cancer, and lung squamous cell carcinoma [95,154]. The majority of these mutations are LOF related to processing phosphorylations, second-messenger binding, or catalysis. Numerous other mutations are in conserved motifs required for catalytic activity [155]. However, there is still a poor understanding of PKC isozyme-specific substrates and the distinct effector role in cancer. Among these unresolved issues is the role of PKC isozymes as tumor promoters versus tumor suppressors [156].

The expression and functions of PKC isozymes in cancerous cells largely depend on the type of cancer from which they originate. For example, PKCδ acts as an antiapoptotic regulator in chronic lymphocytic leukemia (CLL) [157] and as a proapoptotic factor in acute myeloid leukemia (AML) [158], and PKCα shows proliferative functions in several types of cancer [159] but has antiproliferative functions in colon cancer cells [160]. The PKC isoforms signal through multiple pathways and controls the expression of numerous genes relevant for cell-cycle progression, tumorigenesis, and metastatic dissemination, demonstrating variable expression profiles during cancer progression depending on cell types. The first reported cancer-associated mutation in PKC was one in PKCα found in human pituitary tumors [161,162]. While today we better understand the involvement of individual PKCs in various cancer types and in the context of specific oncogenic alterations, PKC contributions to malignancy remain a challenge, and the relevance of individual PKC isozymes in the progression of human cancer, in many cases, is still a matter of controversy.

Skin cancer is the most common cancer in the United States (US), when including melanomas and nonmelanomas, and about one in five Americans will develop skin cancer in their lifetime. Nonmelanomas are more common and include basal cell carcinoma (BCC) and squamous cell carcinoma (SCC), while melanoma is a more serious yet less common type of skin cancer. PKC isozymes exhibit specificities in their signals relative to the development of skin cancer. PKCα is highly abundant in the skin, and its activation is typically associated with increased tumor cell proliferation, invasiveness, and decreased differentiation, which was suggested by studies done with PKCα inhibitors [163,164]. PKCε induces SCC by an inhibition of apoptosis and enhancement of preneoplastic cell proliferation [151,156,165].

Breast cancer is the second most common cancer in women in the US, and about one in three of all new female cancer diagnoses each year are breast cancer. Breast cancer is also the second leading cause of cancer death in women. PKC isozyme expression and localization are tightly controlled during the processes of mammary gland differentiation and involution. Overexpression of several PKC isozymes has been reported in malignant breast tissue and breast cancer cell lines. PKCβ, PKCδ, and PKCη are found mainly in early-stage breast cancers and decrease with increasing stage, while PKCα and PKCζ levels typically increase as the cancer progresses [156,166].

Lung cancer is the third most common cancer in the US. The two key types of lung cancer are nonsmall cell lung cancer (NSCLC), which is the most common lung cancer accounting for about 85% of lung cancer cases, and small cell lung cancer (SCLC), which is the most aggressive form of lung cancer. Lung cancer is by far the leading cause of cancer death, making up almost 25% of all cancer deaths in both men and women. PKCα, PKCε, PKCη, PKCι/λ, and PKCζ are upregulated in lung cancer. Furthermore, overexpression of these PKC isozymes correlates with a worse prognosis in NSCLC patients, mainly due to tyrosine kinase-inhibitor resistance [156,167].

Prostate cancer is the second most common cancer in men and the fourth most common cancer overall. Prostate cancer is the second leading cause of cancer death in American men, behind only lung cancer, and about one man in eight will be diagnosed with prostate cancer during his lifetime. Various PKC isozymes play key roles during the progression of prostate cancer. PKCι/λ was overexpressed in invasive prostate cancer tissue, and its inhibition significantly promoted apoptosis and reduced the proliferation of prostate cancer cells [168], although there are other conflicting reports related to prostate cancer growth and metastasis as well [151,156].

Colorectal cancer (CRC) is the third most common cancer worldwide and accounts for eight percent of all cancer deaths both in men and women in the US. There are many types of CRC, the most common being adenocarcinoma, which develops in the lining of the large intestine (colon) or the distal end of the colon (rectum) and makes up 95% of all colorectal cancer cases. While, in general, the assumption is that increased PKC activation and expression promotes carcinogen-induced tumorigenesis, analysis of PKC gene-expression levels in CRC tissues revealed a downregulation of PKCβ. In addition, activation and overexpression of PKCβII dramatically reduced PKB phosphorylation and consequently reduced cell survival, suggesting that PKCβII has a tumor suppressor role in colon cancer [169].

Kidney cancer is less common but still among the top ten most common cancers in the US. The kidneys are made of a lot of small tubules, called the renal tubules, and most kidney cancers develop from these tubules. There are many types of kidney cancer, including renal cell carcinomas (RCC), transitional cell carcinomas, Wilms tumors, and renal sarcomas. Yet, RCC is the most common type of kidney cancer in adults and certainly the most well studied. Increased PKC activity has been noticed during the development and progression of RCC, and several PKC inhibitors have been found to decrease the invasiveness of aggressive human RCC cell lines, suggesting a proinvasive role for PKC in this context [170,171]. For example, increased PKCζ expression was associated with larger tumor size and worse survival, although PKCζ downregulation produced significant chemoresistance in RCC cell lines [172,173].

Bladder cancer is the second most common genitourinary malignancy in the US, the fourth most common cancer, and three times more common in men than in women [174]. Bladder cancer is a complex disease associated with high morbidity and mortality. Urothelial carcinoma (i.e., transitional cell carcinoma or TCC), is by far the most common type of bladder cancer, accounting for 95% of bladder cancer cases [175,176]. PKCα, PKCβ, PKCδ, PKCε, PKCη, and PKCζ have been studied in bladder cancer cells and tissues. Activated PKCα was increased in bladder cancer cells, and several studies demonstrated a correlation between PKCα activation and bladder cancer cell proliferation, survival, invasion, migration, and drug resistance [177,178,179].

Non-Hodgkin’s lymphoma (NHL) is a group of blood cancers originating from white blood cells (lymphocytes) that generally develop in the lymph nodes and lymphatic tissue. NHL accounts for about four percent of all cancers in the US [180,181]. PKCβ promotes B-cell receptor signaling, enhancing B-cell proliferation and survival, which is critical for B-cell-derived lymphomas [182]. PKCβ also modulates angiogenesis via vascular endothelial growth factor (VEGF), which enhances tumor progression [183] and is associated with worse prognoses in NHL [184]. PKCβ was especially overexpressed in treatment-refractory diffuse large B-cell lymphoma (DLBCL), an aggressive, and the most common, type of NHL [185,186].

Thyroid cancer is more common in women than men, and its incidence continues to rise worldwide [187,188]. A single-point mutation in PKCα (Gly for Asp substitution at position 294) was present in 50% of follicular thyroid neoplasms [189] and was associated with altered subcellular distribution and greater growth potential in rat fibroblasts [190]. Furthermore, a rearrangement in the gene-encoding PKCε was reported in a thyroid follicular carcinoma cell line, suggesting that these signaling proteins may play a role in thyroid tumorigenesis [155,191,192].

Endometrial cancer develops from the endometrium lining of the uterus, and the less common gynecological cancer uterine sarcoma develops from the myometrium muscle wall of the uterus [193]. PKCα plays a fundamental role in endometrial carcinogenesis by regulating endometrial cancer cell proliferation, anchor-independent growth, and xenograft tumorigenesis in animal models [194]. PKCδ is also a critical regulator of apoptosis and cell survival in endometrial cancer cells. There is a decrease in PKCδ expression in endometrial tumors, and endometrial cancer cell lines derived from poorly differentiated tumors exhibited reduced PKCδ levels relative to well-differentiated lines. In this context, decreased PKCδ expression may compromise the ability of cells to undergo apoptosis, perhaps conferring resistance to chemotherapy [195,196].

Pancreatic cancer accounts for about three percent of all cancers in the US and about seven percent of all cancer deaths, and it is the tenth most common cancer worldwide. There are two types of tumors that grow in the pancreas: exocrine and neuroendocrine tumors. More than 90% of all pancreatic tumors are exocrine tumors, and the most common kind of pancreatic cancer is called pancreatic ductal adenocarcinoma (PDAC) [197,198,199]. Several PKC isozymes have been shown to play a role in pancreatic cancer [200,201]. PKCι/λ was highly expressed in human pancreatic cancers, and high PKCι/λ expression predicted poor survival [202]. In addition, overexpression of PKCδ in cells induced a more malignant phenotype in vivo due to enhanced cell proliferation and survival [203].

Protein kinases were among the early and favored targets for precision cancer treatment. Since the PKC family has been well-characterized in numerous cancers, PKC became a particularly sought-after target for anticancer agents. Clinical data reveal reduced or elevated protein levels of PKC isozymes in tumor tissue compared with cognate normal tissue depending on the tissue of origin. For example, decreased levels of PKCβ, PKCδ, PKCε, and PKCη were reported in colon cancer [95], while increased levels were reported for PKCγ in colon cancer [204] and PKCι/λ in pancreatic cancer [202]. An additional level of complexity is that certain PKC isozymes are upregulated or downregulated in different cancers. For example, PKCα is upregulated in bladder, endometrial, and breast cancers, while it is downregulated in colorectal tumors and renal cell carcinomas. Yet, over three decades of clinical trials for various cancers using PKC inhibitors not only failed but, in some cases, worsened patient outcomes [205]. A meta-analysis of five clinical trials for NSCLC revealed worse patient outcomes when PKC inhibitors (enzastaurin, an ATP competitive inhibitor, or aprinocarsen, a PKCα antisense oligonucleotide) were combined with chemotherapy versus chemotherapy alone [205], highlighting the complexity of protein kinase signal transduction and the need for the development of more selective tools for basic research as well as therapeutic application.

## 4. PKC in Cardiovascular Diseases

Cardiovascular diseases are a general term for conditions affecting the heart or blood vessels, and they are the leading cause of death globally, taking an estimated 17.9 million lives each year, representing 32% of all global deaths. Almost half of all adults in the US have at least one form of heart disease, and about 659,000 people in the US die from heart disease each year, which is one in every four deaths per year. On average, one person dies from CVD every 36 s. In the US, CVDs claim more lives each year than all forms of cancer and chronic lower respiratory disease combined. Between 2017 and 2018, total direct and indirect costs of CVD were USD 378 billion, and they accounted for 12% of total US health expenditures during this period, which is more than any other major diagnostic group [206,207].

CVDs include all the diseases of the heart and circulation, yet there are many different types of CVDs, and the underlying mechanisms vary depending on the disease. CVDs have a multifactorial origin, and thus a mechanistic understanding of these diseases has been difficult to achieve because of clinical and biological heterogeneity. Furthermore, there is a strong component of race, gender, and environmental factors in the etiology of CVDs. CVDs include different pathologies, such as heart failure, ischemic heart disease, ischemia–reperfusion injury, arrhythmia, cardiomyopathies, and diseases of blood vessels (vascular diseases) including hypertension, atherosclerosis, and stroke. The pathogenesis of many of these CVDs involves protein kinases signaling.

In the heart, kinases mediate signal-transduction pathways that are vital for cardiac function, cation transport, myocardial metabolism, gene expression, cellular growth, and cell apoptosis. However, the relative importance of individual kinases is not clear, and many highly expressed cardiomyocyte kinases remain unstudied in this context. Protein kinase regulation and function are usually studied in proliferating cells in relation to cancer, for which they are attractive therapeutic targets, as previously discussed. Therefore, it is not surprising that most kinase inhibitors that have been approved or are in development are for the treatment of cancer. While the nature of kinase involvement in cancer is an intrinsic property mainly due to aberrant kinase activity that is a result of mutations, in CVDs, kinase-mediated pathogenesis is more frequently due to enhanced stimulation by activated neurohormonal systems. Nevertheless, the success of kinase inhibitors in cancer treatment has strongly supported their application to the treatment of CVDs as well. Furthermore, kinases that promote cancer are often required for cardiac function, and at least some of these kinase inhibitors have cardiotoxic effects in a significant percentage of patients, suggesting activity in this organ [208,209,210].

PKC isozymes are ubiquitously expressed in all tissues at all times of development, and they have emerged as important regulators of the cardiovascular system. The activation of PKC isozymes triggers various intracellular events influencing multiple physiological processes in the heart, including heart rate, contraction, and relaxation. Therefore, PKC isozymes have emerged as important regulators of cardiac function [211,212]. Many PKC isozymes, including PKCα, PKCβ, PKCγ, PKCδ, PKCε, PKCθ, PKCι/λ, and PKCζ, have been found in the hearts of different animal species [213,214,215]; yet, with the vast amount of studies on animal models, there exist only a few reports available that examined the expression of PKCs in humans. In the human heart, six PKC isoforms are expressed, including PKCα, PKCβ, PKCδ, PKCε, PKCι/λ, and PKCζ, while PKCγ and PKCθ are not present [216,217,218]. Even in other species, the relative content of each PKC isozyme in the heart tissue has been a controversial issue, as evidenced by work on rats [219,220], rabbits [221,222], guinea pigs [223], hamsters [224], and dogs [225].

To add to the complexity of these data, PKC expression levels in the heart change over time. PKCα, PKCβ, PKCε, and PKCζ expression is high in fetal and neonatal hearts but decreased in adult rat hearts [226]. The predominant PKC isozymes in the adult cardiovascular system are PKCα, PKCβ, PKCδ, and PKCε, which participate in various CVDs, including atherosclerosis, hypertension, atrial fibrillation, and cardiac hypertrophy. Interestingly, the activation of PKC isozymes in the cardiovascular system was demonstrated in some cases to have opposing effects. While activation of PKCε protects mitochondrial function prior to ischemic events or during reperfusion by activating aldehyde dehydrogenase 2 (ALDH2), removing toxic aldehyde-lipid peroxidation products [227], inhibiting L-type calcium channel activation, and preventing ventricular arrhythmias associated with ischemia–reperfusion injury [228], PKCδ activation mediates damage mainly by activating mitochondrial pyruvate dehydrogenase kinase, which inhibits pyruvate dehydrogenase and ATP regeneration, resulting in necrosis [229,230,231].

Based on the important role that PKC isozymes have in the cardiovascular system, several PKC inhibitors have been tested in animal models of CVDs. Ruboxistaurin (i.e., LY333531, or arxxant) is an orally active macrocyclic bisindolylmaleimide compound that specifically targets PKCβ, which acts as a competitive inhibitor by interacting with the ATP binding site. Ruboxistaurin has shown promising preclinical results in animal models of diabetic retinopathy and macular edema [232,233], and it is being studied as a systemic treatment for diabetic retinopathy, which is a microvascular complication of diabetes. In various myocardial infarction animal models, ruboxistaurin showed a cardioprotective effect. In a rodent model of myocardial infarction, ruboxistaurin protected against cardiac microvascular ischemia–reperfusion injury and reversed cardiac microvascular barrier dysfunction [234,235]. In a porcine myocardial infarction model, ruboxistaurin partially reversed cardiac remodeling by improving contractility [236].

Studies also suggest that PKCδ activity may promote reperfusion injury after ischemia. PKCδ inhibition during reperfusion can block these effects and protect the heart from further damage. Rottlerin is a polyphenol natural product isolated from the Asian tree *Mallotus philippensis*, which has been reported to selectively inhibit PKCδ translocation and reduce infarct size in a rat model of myocardial infarction [237]. However, there were problems with rottlerin related to a lack of specificity, causing significant off-target effects [238]. An attractive alternative approach is the inhibition of PKCδ with specific peptide allosteric inhibitors that can protect the heart from injury, which we will discuss later.

Atherosclerosis is the thickening or hardening of arteries caused by the buildup of plaque in the inner lining of an artery. When atherosclerosis affects the arteries that carry blood to the heart muscle, the terms coronary artery disease (CAD) or ischemic heart disease (IHD) are used to describe a group of CVDs where the heart arteries cannot deliver enough oxygen-rich blood to the heart. CAD encompasses the most common forms of CVD and leads to conditions called stable angina, unstable angina, myocardial infarction, and sudden cardiac death. About 18 million adults have CAD, and, in 2019, over 350,000 people in the US alone died from complications of CAD. Atherosclerosis is also a chronic inflammatory reaction, and it is the primary cause of myocardial infarction and stroke. Numerous studies have been conducted to investigate the significance of PKC in atherosclerosis. Current research suggests that a major role for PKC in atherosclerosis and restenosis relates to its involvement in programmed cell death (apoptosis), yet the exact role that apoptosis plays in atherosclerotic plaque formation is a matter of considerable debate [239,240].

Heart failure (HF) is a clinical syndrome characterized by an impaired ability of the ventricle (most often the left ventricle) to fill and/or eject blood. Heart failure is an ultimate state that can be caused by various insults or stressors to the heart and is characterized by cardiomyocyte hypertrophy, a reduced number of cardiomyocytes, and cardiac fibrosis. The American Heart Association (AHA) statistical update in 2015 reported that one in nine deaths have heart failure mentioned on the death certificate, and, in 2012, the total cost for heart failure was estimated to be about USD 31 billion. A projection shows that by 2030, the total cost of heart failure will increase to almost USD 70 billion [241]. The PKC family has been identified to play a role in the heart’s response to mechanical, electrical, or neurohumoral stimuli, in the case of disease leading to a maladaptive cardiac system characterized by fibrosis, apoptosis, and cardiac remodeling [144,242]. Levels of PKCα and PKCβ isozymes increase during heart-failure progression [215,243], and their inhibition has shown dramatic protective effects [143,244]. Although the role of PKCδ in the pathogenesis of heart failure is not entirely clear, many studies have confirmed that PKCδ is associated with the occurrence of heart failure, and it is activated in the early stages of pressure overload and heart failure [245]. Reduced cardiac expression of PKCε was found in rabbit left ventricular hypertrophy [246] and was also consistent in human left ventricular heart failure [216].

Hypertension (HTN) is a multifactorial disorder that involves pathological changes in the neuronal, renal, and vascular control mechanisms of blood pressure and may lead to various additional cardiovascular complications [247]; 68 million US citizens, or one in every three adults, have hypertension, which contributes to nearly 1000 deaths a day. In 2009, nearly 350,000 American deaths included hypertension as a primary or contributing cause. Annual costs directly attributable to hypertension are projected to more than double over the next two decades, bringing the projected annual total to USD 200 billion by 2030. An increased expression of PKC in vascular smooth muscle can cause excessive vasoconstriction, narrowing of blood vessels, and trophic vascular changes resulting in an increase in vascular resistance and hypertension [248,249,250]. PKCα overexpression has been implicated in the pathogenesis of hypertension [251,252,253], and an increased level of PKCβ was also demonstrated in a rat model of hypertension-induced cardiac dysfunction by a high-salt diet [254], which was consistent with the analysis of human tissue from heart-failure patients [67,215,216,255]. PKCδ is also involved in promoting hypertension via endothelin-1 signaling [231], and inhibition of PKCε in hypertension-induced heart failure led to reduced pathological remodeling and improved myocardial function [256].

Stroke is another complication of CVD. There are three main types of stroke, including ischemic stroke (85% of strokes), in which a blood clot prevents oxygenated blood from reaching an area of the brain; hemorrhagic stroke, in which a blood vessel ruptures; and transient ischemic attack, in which the blood flow to a part of the brain is inadequate for a brief period of time and normal blood flow resumes after a short amount of time with a resolution of symptoms. Stroke is the number five cause of death and a leading cause of disability in the US; about 800,000 people have a stroke each year, and around one person has a stroke every 40 s [257]. A largely conflicting body of work suggests that PKC is activated and plays a pathogenic role during stroke. In contrast, many studies also report a rapid loss of PKC activity and expression after ischemia, suggesting that PKC is degraded. These conflicting data can be explained by different experimental settings, including animal models, types of primary brain tissue, and the duration and intensities of ischemic–reperfusion injury. Furthermore, there are also likely opposing roles of individual PKC isozymes, as discussed previously [258,259,260,261,262].

Arrhythmias include a broad spectrum of disorders relating to heart rate and rhythm abnormalities. Approximately one in eighteen people, or five percent of the US population, have an arrhythmia, with atrial fibrillation being the most common, affecting about 20% of the general population at some time in their lives [263]. As PKCs play a critical role in the regulation of ion channels, the initiation and propagation of the cardiac action potential and thus cardiac excitability can be dramatically affected by PKC activity, yet conflicting data exist for the role of each specific PKC isozyme [228].

Cardiac arrest is the sudden loss of blood flow throughout the body resulting from the heart not being able to pump blood efficiently. There are more than 356,000 out-of-hospital cardiac arrests annually in the US, nearly 90% of them fatal. One estimate placed the total cost of cardiac arrest in the US at USD 33 billion each year [264,265]. Several PKC isozymes were demonstrated to be involved in various mechanisms leading to neuronal cell death after cardiac arrest, such as neurotransmitter release, regulation of ion channels, and modulation of synaptic function. Therefore, PKC isozymes have been suggested as valuable targets for the modulation of this system. In addition, PKCs have been implicated in both increased damage after cerebral ischemia as well as neuroprotection after ischemic preconditioning [217,266].

Myocardial infarction (MI) occurs when blood stops flowing properly to a part of the heart, and the heart muscle is subsequently injured due to a lack of oxygen supply. Often, myocardial infarction is a late-stage complication of coronary artery disease. Myocardial infarction is caused by persistent ischemia and hypoxia due to the inadequate function of coronary arteries, accompanied by a tremendous amount of subsequent myocardial necrosis. The principal treatment for myocardial infarction has focused on revascularization and reperfusion of blocked arteries [267], although a considerable portion of the cardiac damage leading to morbidity and mortality occurs with inflammation and the release of toxic species following reperfusion of the cardiac muscle. Every year, about 805,000 people in the US have a myocardial infarction. The estimated direct and indirect costs of myocardial infarctions from 2016 to 2017 were about USD 12 billion [268]. Increased expression of PKCα was observed following myocardial infarction [216], and inhibition of PKCβ in a postmyocardial infarction heart failure rat model improved cardiac function and was associated with reduced pathological myocardial remodeling [255]. While both PKCδ and PKCε are activated in the ischemic human heart, the mechanism and exact role of each PKC in the survival of cardiac cells remain unknown and controversial. These two isozymes have both parallel and opposing effects on the heart [269,270], indicating potential danger in the use of nonselective isozyme inhibitors and activators.

## 5. PKC in Other Human Diseases

The PKC family has long been considered to play a critical role in the pathogenesis of numerous human diseases due to the diversity of function and broad specificity of different PKC isozymes [101]. PKC isozymes regulate the phosphorylation of proteins that are associated with metabolic disorders, a cluster of various medical conditions that interfere with the body’s metabolism that are usually caused by genetic defects. Furthermore, PKC activation was demonstrated to be involved in signaling pathways dysregulated in metabolic disorders, expression of growth factors, and intracellular levels of ROS that cause cell damage. Therefore, individual PKC isoforms were explored as potential targets in several animal models of metabolic diseases, and some preliminary preclinical studies have identified individual PKC isoforms as potential targets for treating metabolic diseases [55,56,240,271]. For example, PKCβ is a major regulator of cholesterol and fatty acid metabolism in the liver, which phosphorylates and controls the nuclear translocation of the Farnesoid X receptor (FXR), a key regulator for cholesterol removal [272,273]. PKCβ expression levels increased in mice fed high-fat and high-cholesterol diets, and PKCβ-deficient mice are protected against diet-induced obesity and insulin resistance [274].

Several lines of evidence suggest abnormalities of PKC in various renal diseases as well. The two most studied kidney diseases are diabetic nephropathy, the leading cause of end-stage renal disease, and renal cancer [171]. PKC isozymes mediate renal development during the structuring of the embryological metanephros [275] and mediate inflammation in the mature kidney [276]. Studies on the molecular mechanisms of PKC activation in renal disease indicate that activation of PKC signaling, enhanced polyol signaling, increased oxidative stress, and overproduction of advanced glycation end products lead to hyperglycemia and promote diabetic vascular complications [277,278]. Aberrations in PKC expression and localization have also been implicated in the development of kidney cancer. PKCα, which has tumor-suppressor properties, was decreased in renal cell carcinoma (RCC or hypernephroma), the most common kind of kidney cancer in adults [279].

PKC isozymes have also been implicated in pulmonary disease and were specifically found to mediate lung functions, including permeability, contraction, migration, hypertrophy, proliferation, apoptosis, and secretion [280,281,282]. In many cases, the role of these isozymes in specific lung-disease pathogenesis is complicated and has not been fully defined yet. PKC signaling pathways contribute to the key cellular responses central to asthma [283,284,285], chronic obstructive pulmonary disease (COPD) [286], idiopathic pulmonary fibrosis (IPF) [287], acute respiratory distress syndrome (ARDS) [288,289], and lung cancer [167,290], to mention a few. Many markers of nuclear factor kappa B (NF-κB) activity were elevated in asthma and COPD, and the atypical PKCζ regulates NF-κB by phosphorylating its p65 subunit, which is essential for the transcriptional activity of NF-κB. Abdel-Halim et al. developed a specific PKCζ allosteric inhibitor, MA130, which inhibited the expression of only a subset of NF-κB-dependent genes, suggesting that targeting PKCζ may be more tolerable for the treatment of inflammatory lung disorder compared to global inhibition of NF-κB with various systemic toxicities [286].

PKC isozymes are highly enriched in the brain and play a vital role in regulating both the pre- and postsynaptic aspects of neurotransmission. Interestingly, PKC was discovered and initially characterized in the bovine brain [49,291]. PKC inhibition impairs brain function on multiple levels [292,293,294], as does abnormal PKC activation [295,296,297,298,299]. PKC isozymes influence short-term neuronal signaling (e.g., neurotransmitter release and ion flux), medium-term (e.g., receptor regulation), and long-term (e.g., cell proliferation, synaptic remodeling, and gene expression) mechanisms [300]. PKC is important for learning and memory, and PKC isozymes are classified as one of the cognitive kinases, as they were demonstrated to regulate synaptic transmission and affect substrates involved in information processing and storage (e.g., myristoylated alanine-rich C-kinase substrate (MARCKS), growth-associated protein 43 (GAP-43) and the N-methyl-D-aspartate (NMDA) receptor) [301,302].

As PKCs are ubiquitously expressed in the central nervous system (CNS) and mediate phosphorylation of transporters, ion channels, and GPCRs, isozymes are emerging as actionable targets in neurodegenerative diseases [2,299], including Parkinson’s disease [303,304,305,306,307], dementia [308,309], chronic pain [310,311,312,313], and Alzheimer’s disease [314,315,316,317]. For instance, β-amyloid (Aβ) peptides generated from sequential cleavage of amyloid precursor protein (APP) by β-site APP-cleaving enzyme 1 (BACE1) play a key role in the pathogenesis of Alzheimer’s disease. PKCδ overexpression increases lipopolysaccharide (LPS)-induced expression of nitric oxide synthase and cytokines, resulting in increased BACE1 expression in the brain. PKCδ knockdown or inhibition reduced BACE1 expression and corresponding Aβ levels, while PKCδ overexpression increased BACE1 expression and Aβ generation [318].

PKC isozymes are also involved in the transmission and modulation of pain from the spinal cord to the brain and specific cortical regions [311,312,319,320]. PKCε and PKCα appear to be involved in peripheral nociception [321], while PKCγ is important for central nociception [310]. For example, PKCε modulates nociception by activation of the transient receptor potential vanilloid receptor 1 (TRPV1) ion channel, a nonspecific cation channel activated by capsaicin [322,323,324]. Overall, PKC may serve as a master switch by which TRPV1 integrates heat and tissue damage to produce hyperalgesia [325].

PKC abnormalities have been implicated in the pathophysiology of psychiatric illnesses as well [326]. Evidence accumulated from multiple laboratories demonstrated that stress-induced deficits arise from excessive PKC signaling, which diminishes prefrontal neuronal firing [327]. Classic PKC isozymes are the most abundantly expressed PKCs in the CNS, and novel PKC isozymes also facilitate the release of the neurogenic growth factor neuregulin [328,329]. Mounting evidence points to a key role for PKC signaling in the pathology of bipolar disorder, a chronic and life-threatening disorder [330,331,332,333,334]. The mainstay treatment for bipolar disorder, lithium, exerts major effects on the PKC signaling cascade, for example by decreasing membrane-associated PKC, including isozyme-specific decreases in the classic PKCα and novel PKCε [335].

The PKC family also plays a critical role in skin keratinocyte function, including proliferation, differentiation, and neoplastic transformation, as well as in various dermatological diseases, including several forms of skin cancer [336]. PKC mediates squamous cell carcinoma (SCC) development and progression, characterized by reduced PKCδ expression and rat sarcoma virus (RAS) pathway activation [337]. Further, PKCε overexpression in mice led to the development of papilloma-free highly metastatic SCC [338]. The classic PKCα is also highly abundant in skin and has been shown to regulate the cell cycle and keratin cytoskeleton formation [339]. Downregulation of the atypical PKCζ contributed to tumorigenesis by releasing constraints on the activity of PKB [340]. In addition to skin cancer, PKC isozymes play roles in other inflammatory dermatological diseases, such as psoriasis, a chronic disease that affects the skin and joints [341]. In a small manufacturer-sponsored study, researchers evaluated sotrastaurin (AEB071), an orally administered potent and selective pan-PKC inhibitor, to 32 patients with psoriasis, resulting in a dose-dependent improvement in psoriasis during the two-week treatment period [342].

Several PKC isozymes are also important global mediators of inflammation and immunity. A major therapeutic approach to address inflammation has been to target the activity of kinases such as PKC that regulate the production of various cytokines and other inflammatory mediators. In the immune system, PKCs are involved in regulating signal-transduction pathways that are important for both innate and adaptive immunity, ultimately controlling the expression of key immune genes. Animal studies of PKC reported immune perturbations when each different isozyme was genetically deleted, with the exception of PKCγ, whose expression is restricted to the CNS and PKCι/λ, whose deletion results in embryonic lethality [343]. Interestingly, these defects are distinguishable between the different PKC isozymes, indicating that each isozyme has some unique role in the immune system [57,58,59,344]. In another model, PKC downregulation tempered the LPS-induced inflammatory response [345]. Several PKC inhibitors have shown promising results for the treatment of inflammatory diseases in preclinical in vivo studies: RO 32-0432, a PKC inhibitor that decreased phorbol ester-induced paw swelling in rats [346,347]; SKF 108753, a PKC inhibitor that inhibits the development of hind-paw swelling in an adjuvant-induced arthritis rat model [348,349]; and GÖ 6850, a PKC inhibitor that inhibited phorbol myristate acetate-induced edema, neutrophil influx, and vascular permeability in mice at levels comparable to indomethacin [350,351]. The efficacy of these PKC inhibitors suggests that PKC is a valid potential target in the development of anti-inflammatory agents [78].

PKC also plays a key role in pathogen entry and immune-mediated pathogen clearance. PKC isozymes regulate modifications to the cell membrane and cytoskeleton relevant to host–microbe interactions, including caveolae virus internalization by the host cell and membrane microdomains involved in endocytosis [352]. These mechanisms have been described for the entry of filovirus [353], human enterovirus [354], echovirus [355], human immunodeficiency virus (HIV) [356,357,358,359], rhabdovirus [360], alphavirus [361], poxvirus [362], herpesvirus [363,364], influenza virus [365,366], dengue virus [367], hepatitis B virus (HBV) [132], respiratory syncytial virus (RSV) [368,369], and adenovirus [362,370]. The activity of different PKC isozymes is context sensitive, and these kinases can be positive or negative regulators of macrophage function critical for host defense against infection. Depending on that context, PKC function can differentially affect pathogen virulence for bacteria (e.g., *Escherichia coli* [371], *Staphylococcus aureus* [372], *Listeria monocytogenes* [373], and *Chlamydia trachomatis* [374]), fungi (e.g., *Candida albicans* [375,376,377]), and parasites (e.g., *Leishmania* [378,379]). A specific example can be found in visceral leishmaniasis (i.e., Kala-azar), a fatal disease endemic to many parts of the tropical world in which intracellular Ca^2+^ is elevated. The causative *Leishmania donovani* contains lipophosphoglycan (LPG), resulting in classical PKC activity downregulation along with upregulation of Ca^2+^ independent atypical PKCζ expression [380] upon macrophage infection, which enables the parasites to survive within the host macrophages [381]. Table 1 includes examples of PKC isozymes in human diseases.

## 6. Targeting PKC

The vital role of dysregulated protein kinase activity in the etiology of human disease has fostered major drug-discovery programs over the past 40 years. Yet, without fully understanding the role of each isozyme and the signal transduction that is specific to each disease and organism, it is a challenge to identify appropriate targets for the development of new drugs, which is likely responsible for the failure of many lead compounds [14,385]. Given their many cellular roles, PKC isozymes are undoubtedly sought-after drug targets, but the similarity in their catalytic domains poses significant challenges in designing specific inhibitors. In addition, the complexity of their interactions; the many secondary messenger systems involved; and their cellular-, tissue-, and organism-specific variability make the development of selective modulators extremely difficult [382,386].

There are up to approximately 10,000 different proteins in a typical human cell, with an average length of about 400–450 amino acids. Taking into account the average amino acid frequencies (Ser, 8.5%; Thr 5.7%; Tyr, 3.0%) [387], a total of 17.2% of the amino acids are potentially available for phosphorylation. Therefore, there are about 700,000 potential phosphorylation targets for any given kinase, depending on the localization, activity, conformation, and availability of cofactors. Thus, there are clearly intricate mechanisms that determine the specificity between kinase and substrate, including local and distal interactions and the formation of complexes with scaffold/adaptor proteins [388].

PKC inhibitors are classified according to their sites of interaction within the PKC protein structure. Categories include (i) inhibitors of the catalytic domain, which are directed to either the substrate- or ATP-binding site and mainly consist of ATP-competitive SMKIs; (ii) modulators of the regulatory domain, which mainly target the phospholipid or phorbol ester binding site of the C1 domain, mimicking the binding of DAG; and (iii) inhibitors that disrupt PPIs (iii_a_) at a specific subcellular location or (iii_b_) with a specific substrate or scaffold protein (e.g., PKC and its corresponding RACK). These inhibitors that target PPIs provide an exciting approach to inhibiting PKC isozymes in a selective manner. This can be accomplished by targeting unique regions in each PKC isozyme, usually with peptides derived from the interacting domains of the protein and the binding protein (e.g., anchoring/scaffold protein or substrate), therefore modulating the phosphorylation of individual proteins. While various inhibitory compounds are available, only a few demonstrate specificity for either PKC alone or individual PKC isozymes. For the chemical structure and selectivity of major PKC modulators evaluated in the clinic, see Table 2.

Inhibitors of the catalytic domain. Since the catalytic domain demonstrates the greatest similarity in sequence and structure between PKC isozymes, developing isozyme-selective inhibitors for this site is extremely challenging [389]. Several small-molecule candidates with PKC activity have been identified by high-throughput screening followed by medicinal chemistry optimization or by in silico screening. These molecules usually act by competing with the ATP binding site to block the phosphotransferase activity of their targets. Bisindolylmaleimides are a family of structurally related compounds and are the most studied PKC SMKIs that act as ATP-competitive inhibitors. Bisindolylmaleimides bind to the ATP-binding pocket, stabilizing PKC in the activated conformation, and limiting substrate phosphorylation. Staurosporine, which belongs to the bisindolylmaleimide class, is a prototypical ATP competitor originally isolated in 1977 from the bacterium *Streptomyces staurosporeus*. The compound acts as a broad-spectrum protein kinase inhibitor, including PKC, as well as several other serine/threonine kinases. Based on the native compound, the synthetic bisindolylmaleimide enzastaurin was developed. Enzastaurin is a more selective PKC inhibitor compared to staurosporine, initially believed to selectively inhibit the PKCβ isozyme [390]. However, later studies demonstrated that enzastaurin inhibits other PKC isozymes as well as several additional protein kinases [391].

Modulators of the regulatory domain. The second group of PKC regulators are compounds that target the C1 domain at the regulatory region, mainly by mimicking the binding of the C1 domain to DAG. To better characterize the DAG binding site of the C1 domain [392], a medicinal chemistry study designed potent PKC activators, including DAG-lactones [393], which demonstrate increased isozyme affinity specificity (e.g., compound 57 for PKCα [394] and AJH-836 for novel PKCs [395]) compared to natural DAGs [396]. The best-characterized compound that targets the C1 domain is Bryostatin-1, a partial agonist resulting in PKC autophosphorylation and translocation to the cell membrane [397,398]. Bryostatin-1 is a macrocyclic lactone derived from a marine invertebrate that binds to the regulatory domain of PKC; interestingly, short-term exposure to bryostatin-1 promoted PKC activation, whereas prolonged exposure promoted PKC downregulation [399,400]. Bryostatin-1 has been investigated for its anticancer activity in numerous hematological and solid-tumor cell lines and was found to inhibit proliferation, induce differentiation, and promote apoptosis. Furthermore, phase I and II clinical trials in a wide range of tumors showed promising activity [400]. Nevertheless, as the C1 binding site is highly conserved among PKC isozymes, it is challenging to develop isozyme-selective compounds. Since different isozymes have diverse functions, modulation of PKC signaling by a compound with pan-PKC activity can have vast cellular effects, including adverse effects.

To achieve superior isozyme specificity, two main approaches have been developed, based on the use of antisense oligonucleotides or peptides. Antisense oligonucleotides demonstrated positive results in preclinical models; for example, early studies with antisense inhibition of PKCα demonstrated that it enhanced the antitumor effects of cisplatin in subcutaneously injected H460 cells [401], and additional studies showed that it significantly impaired nonsmall cell lung cancer tumor growth [402]. While this work and other studies provide a rationale for using specific PKC antisense oligonucleotides to reduce PKC in clinical trials, another PKCα antisense oligonucleotide failed in clinical trials for cancer [205]. An alternative approach to the development of ATP-competitive inhibitors is the use of peptides that target allosteric PPI sites (will be discussed in Part II).

## 7. PKC Inhibitors in Clinical Trials

ATP competitive SMKIs have been broadly developed and applied in clinical trials (Table 3). For example, midostaurin (4′-N-benzoylstaurosporine, also PKC412 or CGP 41251) is a staurosporine analog isolated from *Streptomyces staurosporeus*. The compound is an ATP-competitive inhibitor with low inhibitory activity for PKC. Midostaurin inhibits multiple protein kinases, reduces the growth of cancer cells in vitro, and reverses P-glycoprotein-mediated multidrug resistance in cancer cell lines [403]. FMS-like tyrosine kinase 3 (FLT3) mutations are associated with high leukemic burden and poor prognosis in patients with AML. Midostaurin was approved by the FDA in April 2017 for the treatment of newly diagnosed adult AML patients with FLT3-positive mutations, where it was more cost-effective than the previous standard of care [404]. While midostaurin was originally developed as a PKC inhibitor, its success in early trials was mainly due to the inhibition of other tyrosine kinases. Further clinical trials of midostaurin are in progress [405] (ClinicalTrials.gov ID NCT05488613). Enzastaurin is a cyclic bisindolylmaleimide and an ATP competitive inhibitor that targets PKCβ. This compound was tested in several phase I and phase II clinical trials alone and in combination with other drugs, mainly for oncologic indications (e.g., glioma [406], multiple myeloma [407], solid-tumor brain metastasis [408], ovarian carcinoma [409], lymphoma [410,411], breast cancer [412], NSCLC [413,414], glioblastoma [415], prostate cancer [416], ovarian cancer [417], metastatic colorectal cancer [418], and pancreatic cancer [419]). In these trials, enzastaurin alone or in combination failed to show any significant clinical benefit [420]. Other ATP competitive PKC inhibitors include sotrastaurin, a potent and selective pan-PKC inhibitor that exhibits immunosuppressive functions (e.g., inhibition of T-cell activation [421]) but was plagued by side effects and a lack of efficacy for the indications tested. Moreover, another nonselective PKC inhibitor called flosequinan (manoplax) previously used for congestive heart failure was withdrawn from the US market (October 1993) due to increased hospitalization and mortality [422].

While several C1 domain-binding PKC inhibitors (e.g., DAG competitive PKC inhibitors) have been reported, there are no published findings to describe the use of these compounds in clinical trials. An antisense oligonucleotide-targeting PKCα called aprinocarsen (ISIS 3521/LY900003) was evaluated in several clinical trials for recurrent high-grade astrocytomas [423], NSCLC [424,425], ovarian carcinoma [426], prostate cancer [427], metastatic colorectal cancer [428], and NHL [429]. However, aprinocarsen treatment alone or in combination with anticancer drugs failed to improve survival or confer other clinical benefit [420].

**Table 3:** Summary of clinically studied PKC modulators.

Indication	Compound	Proposed Mechanism	Outcome	Regulatory Status	Refs.
Oncology	Bryostatin	Nonselective PKC activator	No benefit	Not approved	[430,431,432,433,434,435,436,437,438,439,440,441,442,443]
	Aprinocarsen	PKCα inhibitor	No benefit	Not approved	[424,427,428,429,444,445]
	Enzastaurin (LY317615)	PKCβ inhibitor	No benefit	Not approved	[407,409,410,446,447,448,449,450]
	Tamoxifen	Nonselective PKC inhibitor at high doses, ER inhibitor	Used in the management of many breast and gynecologic cancers; failed trials for other malignancies	Approved	[451,452,453]
	Midostaurin	Nonselective PKC inhibitor, FLT3 inhibitor	Used in treatment of FLT3-mutated AML	Approved	[454]
	7-Hydroxystaurosporine (UCN-01)	Nonselective PKC inhibitor, CHK1 inhibitor	No benefit	Not approved	[455,456,457]
	PMA	Nonspecific PKC activator	No benefit	Not approved	
	Safingol	PKCβI, PKCδ, and PKCε inhibitor; PI3K inhibitor	No benefit	Not approved	[458,459]
	12-O-tetradecanoylphorbol-13-acetate	Nonselective PKC activator	No benefit, severe side effects	Not approved	[460]
Diabetes mellitus	Ruboxistaurin(LY333531)	PKCβ inhibitor	Improved diabetic retinopathy but not nephropathy in early studies, minimal effect on neuropathy	Not approved	[461,462,463,464,465,466,467,468,469]
Cardiology	Delcasertib (KAI-9803)	PKCδ inhibitor	No benefit	Not approved	[470,471,472]
	Flosequinan	Nonselective PKC inhibitor	Increased hospitalizations and HF mortality; early study termination	Withdrawn	[422,473]
	Volatile anesthetics	PKCε activator	Reduced troponin I, inotrope requirements, and length of hospitalization	Approved for other indications	[474,475,476,477,478]
	Adenosine	PKCε activator	Reduced MI, mortality, vasopressor requirements	Approved for other indications	[479,480,481]
	Acadesine	PKCε activator, AMPK activator	No reduction in death, MI, or stroke	Not approved	[482,483,484]
Bipolar disorder	Endoxifen	Tamoxifen metabolite with four-fold increased PKC inhibition	Improved time to remission	Not approved	[485]
Nociception	KAI-1678	Inhibits PKCε translocation	No benefit	Not approved	[486,487]
Inflammation	Sotrastaurin (AEB071)	Nonselective PKC inhibitor	Worse outcomes in transplant rejection, no benefit in malignancy or autoimmune trials	Not approved	[488,489,490,491]
Alzheimer’s Disease	Bryostatin	Nonselective PKC activator	Primary endpoint was not significant	Not approved	[492]

PKC, protein kinase C; HF, heart failure; MI, myocardial infarction; ER, estrogen receptor; AMPK, AMP-activated protein kinase; FLT3, fms-like tyrosine kinase 3; AML, acute myeloid leukemia; CHK1, checkpoint kinase 1; PMA, phorbol 12-myristate 13-acetate; PI3K, phosphoinositide 3-kinase. Adapted from refs. [382,420].

## 8. Summary

In this review, we discuss the extensive role PKC plays in cancer and CVDs, making these proteins highly sought-after therapeutic targets. We also emphasize the specific PKC isozymes related to specific conditions in these domains. The last section summarizes the current molecules (mainly SMKIs) used in the clinic as well as recent clinical trials that have been conducted. In particular, we discuss tamoxifen and midostaurin, which are both clinically active PKC inhibitors. Tamoxifen (Nolvadex) is a nonsteroidal antiestrogen used to treat estrogen-receptor-positive breast cancers, and midostaurin (Rydapt or Tauritmo) is a multitarget kinase inhibitor for the treatment of adult patients with newly diagnosed acute myeloid leukemia (AML). While both medications are available for human use, neither of them inhibits PKC selectively. In the subsequent article, we will elaborate on additional therapeutic approaches for PKC, focusing on targeting allosteric sites and their unique advantages.

## Figures and Tables

**Table 1:** PKC isozymes in human disease.

Disease	PKC Isozyme	Associated Pathology
Cancer	PKCα	Proliferation; invasion; metastasis; RCC tumor progression; mutated in thyroid cancer; SCC tumorigenesis and progression; elevated in late-stage breast cancer; upregulated in lung cancer; activated in bladder cancer
	PKCβ	Angiogenesis; invasion; elevated in early-stage breast cancer; downregulated in CRC; PKCβII tumor suppressor in CRC; promotes angiogenesis in NHLs and DLBCL
	PKCδ	Angiogenesis; apoptosis in endometrial cancer; elevated in early-stage breast cancer; downregulated in CRC
	PKCγ	Increased in CRC
	PKCε	Oncogene; proliferation; metastasis; chemotherapy resistance; mutated in thyroid cancer; SCC tumorigenesis, progression, and metastasis; keratinocyte cytoskeleton structure; upregulated in lung cancer; downregulated in CRC
	PKCη	Glioblastoma tumorigenesis; proliferation; radiation resistance; elevated in early-stage breast cancer; upregulated in lung cancer; downregulated in CRC
	PKCθ	Gastrointestinal stromal tumorigenesis
	PKCι/λ	Oncogene; upregulated in lung cancer; promotes prostate cancer invasion; conflicting roles in prostate cancer metastasis; elevation associated with poor outcomes in PDAC
	PKCζ	Elevated in late-stage breast cancer; upregulated in lung cancer; promotes RCC tumor progression
Diabetes mellitus	PKCβ	Obesity; glucose transport; insulin resistance; cholesterol and fatty acid metabolism; PKCβII late-stage vascular complications
	PKCδ	Islet cell function
Ischemic heart disease	PKCβ	Pathologic postischemic cardiac remodeling
	PKCδ	ROS production; apoptosis; necrosis; postischemic injury
	PKCε	Mitochondrial function; proteasome function; ALDH2 activation
Heart failure	PKCα	Reduced contractility; β-adrenergic receptor uncoupling
	PKCβII	Decreased proteasome function; calcium dysregulation; conflicting roles in hypertrophy; conflicting roles in contractility
	PKCδ	Abnormal activation in early HF
	PKCε	Fibrosis; inflammation; depleted in late-stage HF
	PKCζ	Generation of active NADPH oxidase; Reduced basal and TGF-βI induced fibroblast proliferation; increased MMP1,3,9 release
Hypertension	PKCα	Elevated in hypertension
	PKCβ	Elevated in hypertension
	PKCδ	Elevated in hypertension
	PKCε	Translocation and increased hypertrophic cellular growth
Stroke	PKCδ	Mitochondrial fission; ROS production; blood–brain barrier dysfunction
	PKCε	Cytoprotective; cerebral perfusion
Neurodegeneration	PKCδ	Inflammation; neuronal cell death in Parkinson’s; β-amyloid formation in Alzheimer’s disease
	PKCα	Associated with AD, promoting synaptic loss
	PKCγ	Associated with SCA, promoting neuronal death
Bipolar disorder	PKCα	Abnormal gene expression
	PKCε	Abnormal neuronal transmission
Nociception	PKCα	Peripheral nociception signaling
	PKCγ	Dorsal root ganglia signal transmission
	PKCε	Spinal cord signal transmission; peripheral nociception signaling
Inflammation	PKCδ	B-cell development; keratinocyte proliferation and dysregulated angiogenesis in psoriasis; eosinophil activation in asthma
	PKCθ	T-cell responses; airway inflammation; joint inflammation
	PKCζ	Lung inflammation

RCC, renal cell carcinoma; HF, heart failure; SCC, squamous cell carcinoma; CRC, colorectal cancer; PDAC, pancreatic ductal adenocarcinoma; NHL, non-Hodgkin’s lymphoma; DLBCL, diffuse large B cell lymphoma; AD, Alzheimer’s disease; SCA, Spinocerebellar ataxia. Adapted from [89,101,211,382,383,384].

**Table 2:** Prototypical PKC inhibitors used in the clinic.

Compound Name	Structure	Indication(s)	Target Domain	Mechanism	Isozyme Selectivity
Midostaurin	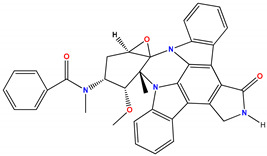	Acute myeloid leukemia	Catalytic domain	Competitive inhibitor of ATP binding site	Multiple protein kinases
Riluzole	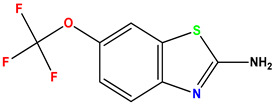	Amyotrophic lateral sclerosis	Catalytic domain	Competitive inhibitor of ATP binding site	PKCα and other kinases
Curcumin	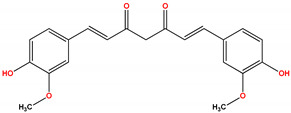	Not approved; used as nutraceutical for ulcerative colitis and other inflammatory conditions	Regulatory domain	C1 inhibitor	PKCα
Resveratrol	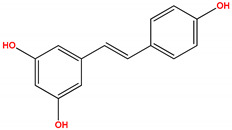	Not approved; ongoing studies in cancer, cardiovascular disease, neurodegeneration, and aging	Regulatory domain	C1 inhibitor	PKCα; PKCβ; PKCε

Adapted from [101,382].
